# Vaginal microecological diversity and Ureaplasma urealyticum co-infection in high-risk HPV infection: a cross-sectional study

**DOI:** 10.3389/fcimb.2026.1851005

**Published:** 2026-06-29

**Authors:** Nanqiu Peng, Yiyi Zhang, Li He, Li Xie

**Affiliations:** 1 College of Medical Technology, Shanghai University of Medicine & Health Sciences, Shanghai, China; 2 Department of Research, Zhoupu Hospital Affiliated to Shanghai University of Medicine & Health Sciences, Shanghai, China; 3 Department of Quality Management, Xuhui District Center for Disease Control and Prevention (Xuhui District Health Supervision Institute), Shanghai, China; 4 Clinical Laboratory, Wusong Central Hospital, Shanghai, China; 5 Wusong Hospital, Zhongshan Hospital Affiliated to Fudan University, Shanghai, China

**Keywords:** bacterial richness, high-risk human papillomavirus (HR-HPV), host-microbe interaction, Ureaplasma urealyticum, vaginal microecology, viral load

## Abstract

**Background:**

Persistent high-risk human papillomavirus (HR-HPV) infection is a key driver of cervical carcinogenesis. Vaginal microecology acts as a host defense barrier, and its dysbiosis may affect HPV clearance. The interplay among microbial diversity, viral load, and sexually transmitted pathogens remains unclear.

**Methods:**

This cross-sectional study enrolled 330 women from a gynecological clinic in Shanghai (December 2025 – February 2026). HR-HPV genotyping and viral load were determined by fluorescence quantitative PCR. Vaginal culturable bacteria were identified using MALDI-TOF MS, and culturable bacterial richness (Sobs index) were calculated. Routine vaginal parameters and sexually transmitted pathogens (Chlamydia trachomatis, Neisseria gonorrhoeae, Ureaplasma urealyticum [UU]) were assessed. Microecological characteristics were compared between HR-HPV-positive and -negative groups, with subgroup analyses within the positive group by viral load, genotype, and single/multiple infection.

**Results:**

The HR-HPV positivity rate was 43.94% (145/330), with HPV52, HPV53, and HPV16 being predominant, and the highest rate in women >50 years. The HR-HPV-positive group had significantly higher bacterial richness (Sobs index) than the negative group (all P<0.05). Among positive patients, those with high viral load (logarithm >4) had significantly higher Sobs index than those with low viral load (P < 0.05). Proportions of patients with pH>4.5, positive H_2_O_2_, positive leukocyte esterase, and Lactobacillus count <20 were significantly higher in the positive group (all P<0.05). UU positivity was significantly higher in the positive group (78.1% vs. 52.3%, P<0.05), while CT and NG showed no significant differences. Within the positive group, no significant differences in diversity or routine parameters were observed across subgroups (all P>0.05).

**Conclusion:**

HR-HPV infection is associated with elevated culturable bacterial richness and dysbiosis; high viral load correlates with more pronounced microbial alterations. UU co-infection is positively associated with HR-HPV. These findings support incorporating vaginal microecological assessment and UU screening into HR-HPV management strategies.

## 1 Introduction

Cervical cancer is a common gynecological malignancy and ranks fourth in global cancer incidence among women, predominantly affecting those over 50 years of age. In 2022, there were approximately 660,000 new cases and 350,000 deaths worldwide (Bray et al., 2024). In China, the corresponding figures for 2023 were approximately 109,741 new cases and 59,060 deaths, placing the country second globally in cervical cancer burden (Bruni et al., [[NoYear]]). Persistent infection with high-risk human papillomavirus (HR-HPV) is recognized as a critical etiological factor in the development of cervical lesions and subsequent cervical cancer. The virus is transmitted primarily through sexual contact and poses a substantial threat to women’s health.

The progression of cervical lesions following HR-HPV infection is influenced by multiple factors, among which the vaginal microecology is considered a key modulator. Under normal conditions, the vaginal microecological environment is dominated by Lactobacillus species, which maintain a low pH and support a low-diversity ecosystem that helps resist pathogen invasion, suppress opportunistic bacteria, facilitate local immunity, and resolve inflammation. Disruption of this balance—characterized by a reduction in lactobacilli, overgrowth of pathogens, and microbial colonization—impairs the local immune defense, compromises HPV clearance, and promotes the development of vaginal inflammatory conditions (Chen et al., 2021; Lin et al., 2022).

In addition, HPV genotype, viral load, and infection status are closely associated with cervical lesion progression. Previous studies have suggested that high viral load correlates with the progression of cervical intraepithelial neoplasia (CIN); however, the precise relationship between viral load and the vaginal microecology remains incompletely understood (Sivars et al., 2023).

To address this gap, the present study enrolled women attending the gynecological outpatient clinic of Wusong Central Hospital, Baoshan District, Shanghai, between December 25, 2025, and February 6, 2026. HPV genotyping was performed, and participants were categorized into HR-HPV-positive and HR-HPV-negative groups. Vaginal bacterial cultures were conducted, and microbial diversity indices were calculated for comparison between groups. Within the HR-HPV-positive group, subgroup analyses were performed according to viral load, genotype, and infection status to evaluate associations with vaginal discharge parameters and sexually transmitted diseases (STD). The study aims to elucidate the correlation between vaginal microecological indicators and HR-HPV infection, thereby providing an evidence-based reference for the clinical prevention and early intervention of HR-HPV infection.

HPV is a small, non-enveloped, double-stranded DNA virus belonging to the Papillomaviridae family. Over 200 HPV genotypes have been identified, with transmission occurring primarily through sexual contact, as well as through indirect contact and mother-to-child transmission. HPV infection may result in benign or malignant proliferative lesions of the skin and mucous membranes (Brody, 2012).

Based on their oncogenic potential, HPV types are classified as low-risk (LR) or high-risk (HR). LR types, predominantly HPV6 and HPV11, are associated with benign conditions such as anogenital warts. HR types, including HPV16, HPV18, HPV31, HPV33, HPV45, HPV52, and HPV58, are causally linked to malignancies of the cervix, anus, oropharynx, and other sites. Following HR-HPV infection, abnormal expression of the E6 and E7 oncoproteins suppresses host tumor suppressor gene function, leading to uncontrolled cell proliferation, genomic instability, and ultimately epithelial dysplasia. The risk of cervical lesion progression is substantially higher for HR-HPV than for LR-HPV infections (Ramirez-Flores et al., 2016).

In most cases, HPV infection is self-limiting and can be cleared within one year by the host immune system, depending on factors such as genotype, viral load, infection site, and immune status. However, a subset of individuals fails to clear the virus and develops persistent infection, placing them at elevated risk for cervical cancer. Progression to CIN occurs, with grades CIN1, CIN2, and CIN3, the latter two representing high-grade precancerous lesions. Without intervention, these may progress to invasive carcinoma over a period of years to decades, providing a critical window for screening and early treatment. Studies indicate that the positivity rate of HPV E6/E7 mRNA is positively correlated with the severity of cervical lesions (Hui et al., 2021); thus, detection of E6/E7 mRNA may serve as an indicator of high-grade cervical lesions.

Vaccination represents a primary prevention strategy against HPV infection and related malignancies. Current prophylactic HPV vaccines are based on virus-like particles assembled from the L1 capsid protein, which do not contain viral nucleic acids and are both safe and immunogenic. Three main vaccines are available: bivalent (Cervarix), quadrivalent (Gardasil), and nonavalent (Gardasil 9), covering 9–45-year-olds. The bivalent vaccine targets HPV16 and HPV18, conferring protection against the majority of cervical cancers. The quadrivalent vaccine additionally covers HPV6 and HPV11, thereby reducing the incidence of genital warts. The nonavalent vaccine offers broader protection by covering nine HPV types. China has initiated an accelerated action plan for cervical cancer elimination, gradually integrating HPV vaccination into the national immunization program for school-aged girls, while promoting free cervical cancer screening and standardized treatment of precancerous lesions. This three-tiered prevention strategy—vaccination, screening, and treatment—is steadily advancing toward the World Health Organization’s cervical cancer elimination goals.

The vaginal microecology constitutes an essential physical and immunological barrier of the female reproductive tract, comprising a dynamic ecosystem of resident microbiota, endocrine regulation, local immunity, and anatomical structures. Under healthy conditions, Lactobacillus species predominate, coexisting with a limited number of other microorganisms such as Streptococcus, Staphylococcus, Corynebacterium, Escherichia coli, Mycoplasma, and Candida species. This complex ecosystem operates through mutual constraints and interactions, and its stability is critical to female reproductive health (Tsementzi et al., 2025). The central function of the vaginal microecology is the maintenance of local defense. Lactobacillus metabolizes glycogen from vaginal epithelial cells to produce lactic acid, maintaining the vaginal pH between 3.8 and 4.5. This acidic environment inhibits the overgrowth and adhesion of pathogenic and opportunistic bacteria, prevents invasion by exogenous pathogens, and establishes a “biological barrier”—a mechanism known as the vaginal “self-cleansing” process, representing the first line of defense against reproductive tract infections.

The vaginal microecology is vulnerable to disruption by factors such as age, hormonal status, sexual activity, hygiene practices, antibiotic use, vaginal douching, pregnancy, and immune status (Žukienė et al., 2025). When the balance is disturbed, Lactobacillus abundance declines, pH rises, and pathogenic bacteria, anaerobes, and Candida species overgrow, leading to various forms of vaginitis with symptoms including abnormal discharge, pruritus, and malodor. Recent studies have demonstrated that vaginal microecological dysbiosis is not only closely associated with reproductive tract infections but also linked to multiple gynecological conditions and adverse reproductive outcomes. Persistent dysbiosis may contribute to recurrent infections and increase the risk of persistent HPV infection, CIN, and cervical cancer (Chen et al., 2024).

Ureaplasma urealyticum (UU), Chlamydia trachomatis (CT), and Neisseria gonorrhoeae (NG) are the three most common pathogenic microorganisms in the female urogenital tract. They are primarily transmitted sexually, but can also be spread vertically from mother to child or indirectly via contaminated fomites. Infections with these pathogens can cause reproductive tract infections, trachoma, urogenital lesions, suppurative inflammation, and adverse pregnancy outcomes (Cai et al., 2020; Liu et al., 2024; Kristensen et al., 2025).

Collectively, UU, CT, and NG infections are closely associated with HPV infection and serve as important risk factors for persistent HPV infection and cervical lesion progression. Studies have shown that co-infection with HPV and UU, CT, or NG may worsen disease severity, and HR-HPV-positive patients with concurrent UU, CT, or NG infection exhibit more severe cervical lesions and faster progression compared to those with HPV infection alone (Silva et al., 2014; Huang et al., 2020; Swase et al., 2025). Therefore, in the context of cervical cancer prevention, screening and appropriate treatment of UU, CT, and NG, alongside HPV and cervical lesion assessment, are essential components of preserving vaginal microecological balance and interrupting persistent HPV infection.

## 2 Materials and methods

### 2.1 Study population

A total of 330 women who attended the gynecological outpatient clinic of Wusong Central Hospital, Baoshan District, Shanghai, between December 25, 2025, and February 6, 2026, were enrolled.

The inclusion criteria include (1) History of sexual intercourse, with no intercourse within 24 hours prior to sampling, (2) Complete medical records, (3) No use of medications affecting the vaginal microecology within 3 days before sampling, (4) No history of radiotherapy or chemotherapy, (5) No cervical treatments (e.g., conization, cryotherapy) within the past 6 months, (6) No liver or kidney dysfunction. While the exclusion criteria include (1) Pregnancy or lactation, (2) Current or prior treatment for other malignancies, (3) History of AIDS, (4) History of hematologic or autoimmune diseases, (5) Menstrual or abnormal bleeding phase, (6) History of diabetes, hypertension, or heart disease.

### 2.2 Experimental methods

#### 2.2.1 Specimen collection

All samples were collected by gynecologists using sterile procedures.

For cervical cytology, patients were placed in the lithotomy position, and the vulva was routinely disinfected. A vaginal speculum was used to expose the cervix, and excess mucus was gently removed from the external os using a sterile swab. A cytobrush was inserted approximately 1.5 cm into the cervical canal and rotated clockwise three times to collect cervical epithelial cells. The brush was slowly withdrawn, and the head was placed into a centrifuge tube containing cell preservation solution. The handle was broken off, the cap was tightened, and the sample was immediately sent to the laboratory.

For vaginal discharge and STD samples, the cervix and vagina were exposed with a speculum. Sterile swabs were inserted into the posterior vaginal fornix and gently rotated to obtain sufficient secretions, with visible secretions on the swab considered adequate. Swabs were placed into plastic tubes containing specific dilution solutions or preservation tubes.

#### 2.2.2 HPV detection

##### 2.2.2.1 Nucleic acid extraction

Magnetic bead-based nucleic acid extraction kits (Jiangsu Bioperfectus Technologies Co., Ltd.) and an SSNP-9600A automated extractor were used. Samples (200 μL) were added to wells in columns 1 and 7 of the reagent plate, and extraction was performed according to the instrument protocol.

##### 2.2.2.2 HPV testing

The Shanghai Hongshi SLAN-96S real-time PCR system and fluorescence PCR-based HPV genotyping kits (Jiangsu Bioperfectus Technologies Co., Ltd.) were used to detect 17 high-risk HPV types. Primers and probes targeting the L1 region were labeled with FAM, HEX, and ROX. Following PCR amplification, results were automatically interpreted by the instrument.

##### 2.2.2.3 Diagnostic criteria

HR-HPV-negative was defined as absence of detectable HR-HPV; HR-HPV-positive was defined as detection of HR-HPV. Single infection was defined as detection of one genotype; multiple infection was defined as detection of two or more genotypes (**Table 1**).

**Table 1:** Fluorescent probes for HPV genotyping.

Probe label	HPV types
FAM	HPV16, HPV59, HPV33, HPV56, HPV68, HPV82
HEX	HPV18, HPV66, HPV58, HPV52, HPV51
ROX	HPV31, HPV53, HPV45, HPV35, HPV39, HPV73

#### 2.2.3 Vaginal bacterial culture, identification, and diversity calculation

##### 2.2.3.1 Culture

In a biosafety cabinet, 10 μL of the cervical cytology specimen was inoculated onto a blood agar plate and streaked in four quadrants. Plates were incubated at 36.5 °C with 6.5% CO_2_ for 3 days.

##### 2.2.3.2 Identification

Bacterial species were identified using the Autof MS1000 mass spectrometer (Autobio) following standard operating procedures. MALDI-TOF MS generated protein fingerprint spectra (m/z 2000~20,000 Da), which were compared with the database. Scores ≥9.0 were considered confident species-level identification; 6.0~9.0 indicated genus-level identification; <6.0 indicated unreliable results. Scores ≥6.0 were accepted.

##### 2.2.3.3 Diversity calculation

Based on species presence/absence data (each detected species counted as abundance 1), Sobs index (observed species richness) was calculated. Because only culturable bacteria were analyzed and abundance was not quantified, Ace index equaled Sobs index, and Shannon/Simpson indices were not reported in the main manuscript (see **Supplementary Table 1**).

#### 2.2.4 Routine vaginal discharge testing

A bacterial vaginosis kit (dry chemistry) and an automated analyzer were used. Vaginal microecological parameters were assessed as follows:

1. Cleanliness grade: Grade I–II indicated normal; III–IV indicated abnormality.
2. pH: Values >4.5 were considered indicative of dysbiosis.
3. Hydrogen peroxide (H_2_O_2_): Negative indicated normal Lactobacillus function; positive indicated dysfunction.
4. Leukocyte esterase: Positive indicated inflammation.
5. Sialidase: Positive indicated high risk of bacterial vaginosis.
6. Lactobacillus quantification: Counts were averaged from five high-power fields and multiplied by 5. Abundance was graded: 1+: <5; 2+: 5~20; 3+: 20~150; 4+: >150. Grades 3+~4+ were considered normal; 1+~2+ were considered abnormal.
7. Routine vaginal discharge and STD tests were performed based on clinical indications; thus sample sizes vary across analyses.

#### 2.2.5 STD detection

##### 2.2.5.1 Nucleic acid extraction

Manual extraction was performed using kits (Chaozhou Kaipu Biotechnology) according to the manufacturer’s instructions.

##### 2.2.5.2 PCR detection

A multiplex fluorescence PCR assay (Chaozhou Kaipu Biotechnology) was used to detect CT, NG, and UU. Amplification was performed on a SLAN-96S real-time PCR system, and results were interpreted based on cycle threshold values.

##### 2.2.5.3 Diagnostic criteria

Positivity for CT, NG, and UU was recorded.

#### 2.2.6 Statistical analysis

SPSS version 27.0 was used for data analysis. Categorical variables were expressed as frequencies (percentages) and compared using the χ^2^ test. Continuous variables were tested for normality; none followed a normal distribution and were expressed as medians (interquartile range). The Mann–Whitney U test was used for between-group comparisons. Statistical significance was set at P < 0.05. Multivariable logistic regression was performed with HR-HPV positivity as dependent variable; independent variables included age (>50 vs. ≤50), pH (>4.5 vs. ≤4.5), Lactobacillus count (≤20 vs. >20), and UU positivity.

## 3 Results

### 3.1 Age distribution

The overall age range was 17~79 years. The HR-HPV-positive group (n = 145) ranged from 17 to 73 years, and the HR-HPV-negative group (n = 185) from 22 to 79 years. Among HPV-positive women >50 years, 59 (89.4%) were postmenopausal. Age distribution did not differ significantly between the two groups (P > 0.05). However, stratified analysis showed that the HR-HPV positivity rate increased with age, with the highest rate (45.5%) observed in women >50 years (P < 0.05) (**Table 2**).

**Table 2:** Comparison of age stratification between HR-HPV-negative and HR-HPV-positive groups [n (%), M (Q1, Q3)].

Characteristic	HR-HPV-negative (n=185)	HR-HPV-positive (n=145)	Z/χ^2^	P value
Age (years)	44 (33,55)	48 (35,59)	1.126	0.250
Age stratification			7.320	0.026
<30 years	24 (13.0%)	22 (15.2%)		
30~50 years	100 (54.1%)	57 (39.3%)		
>50 years	61 (33.0%)	66 (45.5%)		

Age was compared using the Mann–Whitney U test, expressed as median (interquartile range). Age stratification was compared using the χ^2^ test, expressed as n (%).

### 3.2 HR-HPV infection characteristics

Fifteen HR-HPV genotypes were identified in 145 positive cases. Single infection was present in 106 cases (73.10%), with the three most common types being HPV52 (22.64%), HPV53 (11.32%), and HPV16 (9.43%). Multiple infections were present in 39 cases (26.90%) (**Table 3**).

**Table 3:** Distribution of HR-HPV genotypes.

HPV genotype	Number of cases (n)	Detection rate (%)
Single infection		
16	10	9.43
18	3	2.83
31	3	2.83
33	2	1.89
39	6	5.66
45	2	1.89
51	9	8.49
52	24	22.64
53	12	11.32
56	5	4.71
58	9	8.49
59	2	1.89
66	10	9.43
68	8	7.55
73	1	0.95
Subtotal	106	73.10
Multiple infection		
Including HPV16	6	15.38
Excluding HPV16	33	84.62
Subtotal	39	26.90

Multiple infection was defined as the detection of two or more genotypes.

### 3.3 Association between culturable bacterial richness and HR-HPV infection

The Sobs index (species richness) was significantly higher in the HR-HPV-positive group than in the negative group (P < 0.05) (**Table 4**). Raw data for Shannon and Simpson indices, which under our equal-abundance assumption reflected presence/absence rather than true diversity, are provided in **Supplementary Table 1**.

**Table 4:** Comparison of culturable bacterial richness (Sobs index) between HR-HPV-negative and HR-HPV-positive groups [M (Q1, Q3)].

Group	n	Sobs index	Z	P value
HR-HPV-Negative	185	2 (1.0, 3.0)	4.639	<0.001
HR-HPV-Positive	145	2 (2.0, 3.0)		

Sobs index, Ace index (due to equal−abundance assumption). Mann–Whitney U test.

### 3.4 Culturable bacterial richness in HR-HPV-positive subgroups

Among HR-HPV-positive patients, those with high viral load (logarithmic value >4) had significantly higher Sobs index than those with low viral load (P < 0.05). No significant differences in Sobs index were observed between single and multiple infections or between common and other genotypes (all P > 0.05) (**Tables 5**–**7**).

**Table 5:** Comparison of Sobs index between low and high viral load in HR−HPV−positive patients [M (Q1, Q3)].

Viral Load	n	Sobs index	Z	P value
Low (log ≤ 4)	55	2 (2.0, 3.0)	2.212	0.027
High (log > 4)	90	2 (2.0, 4.0)		

**Table 6:** Comparison of Sobs index between common and other HPV genotypes in single infections [M (Q1, Q3)].

Genotype group	n	Sobs index	Z	P value
Common (16,18,52,53,58)	58	2 (2.0, 3.0)	-0.786	0.432
Other types	48	3 (2.0, 3.0)		

**Table 7:** Comparison of Sobs index between single and multiple HR−HPV infections [M (Q1, Q3)].

Infection status	(n=106)	(n=39)	Z	P value
Single Infection	106	2 (2.0, 3.0)	-0.040	0.968
Multiple Infection	39	2 (2.0, 4.0)		

### 3.5 Vaginal microecological parameters and STD in relation to HR-HPV infection

Among participants with available vaginal discharge data (117 negative, 63 positive), the proportions of patients with pH > 4.5, positive H_2_O_2_, positive leukocyte esterase, Lactobacillus count ≤20, and cleanliness grade III–IV were significantly higher in the HR-HPV-positive group than in the negative group (all P < 0.05). No significant difference was found for sialidase positivity (P > 0.05) (**Table 8**).

**Table 8:** Comparison of vaginal microecological parameters between HR-HPV-positive and HR-HPV-negative groups [n (%)].

Parameter	HR-HPV-negative (n=117)	HR-HPV-positive (n=63)	χ^2^	P value
Cleanliness grade			5.007	<0.05
Grade I–II	81 (69.2)	33 (52.4)		
Grade III–IV	36 (30.8)	30 (47.6)		
pH			5.689	<0.05
≤4.5	23 (19.7)	4 (6.3)		
>4.5	94 (80.3)	59 (93.7)		
Hydrogen peroxide (H_2_O_2_)			4.593	<0.05
Negative	31 (26.5)	8 (12.7)		
Positive	86 (73.5)	55 (87.3)		
Leukocyte esterase			6.847	<0.05
Negative	92 (78.6)	38 (60.3)		
Positive	25 (21.4)	25 (39.7)		
Sialidase			1.643	>0.05
Negative	114 (97.4)	63 (100)		
Positive	3 (2.6)	0 (0.0)		
Lactobacillus count			6.300	<0.05
>20	54 (46.2)	17 (27.0)		
≤20	63 (53.8)	46 (73.0)		

Bold values indicate statistical significance (P < 0.05) as per standard formatting.

Among participants with available STD data (44 negative, 32 positive), UU positivity was significantly higher in the HR-HPV-positive group (78.1%) than in the negative group (52.3%, P < 0.05), while CT and NG positivity did not differ significantly (P > 0.05) (**Table 9**).

**Table 9:** Comparison of sexually transmitted pathogens between HR-HPV-positive and HR-HPV-negative groups [n (%)].

Pathogen	HR-HPV-negative (n=44)	HR-HPV-positive (n=32)	χ^2^	P value
Chlamydia trachomatis (CT)			0.703	>0.05
Negative	42 (95.5)	29 (90.6)		
Positive	2 (4.5)	3 (9.4)		
Neisseria gonorrhoeae (NG)			/	/
Negative	44 (100.0)	32 (100.0)		
Positive	0 (0.0)	0 (0.0)		
Ureaplasma urealyticum (UU)			5.321	<0.05
Negative	21 (47.7)	7 (21.9)		
Positive	23 (52.3)	25 (78.1)		

Bold values indicate statistical significance (P < 0.05) as per standard formatting.

To assess potential selection bias, we compared included vs. excluded patients for each analysis. For vaginal discharge data (180 included), excluded patients (n=150) did not differ significantly in age, HPV positivity, or genotype distribution (all P>0.05). For STD data (76 included), excluded patients (n=254) similarly showed no significant differences (**Supplementary Table 2**).

### 3.6 Vaginal microecological parameters in HR-HPV-positive subgroups

Within the HR-HPV-positive group, no significant differences in any vaginal microecological parameters were observed across subgroups based on viral load, genotype, or single/multiple infection status (all P > 0.05) (**Table 10**).

**Table 10:** Comparison of vaginal microecological parameters within the HR-HPV-positive group according to viral load, genotype, and infection status [n (%)].

Parameter	High viral load (n = 35)	Low viral load (n = 28)	χ^2^	Common genotypes (n = 23)	Other genotypes (n = 25)	χ^2^	Single infection (n = 48)	Multiple infection (n = 15)	χ^2^	P value
Cleanliness grade			0.716			0.298			1.210	>0.05
Grade I–II	20 (57.1)	13 (46.4)		12 (52.2)	15 (60.0)		27 (56.3)	6 (40.0)		
Grade III–IV	15 (42.9)	15 (53.6)		11 (47.8)	10 (40.0)		21 (43.8)	9 (60.0)		
pH			0.654			1.920			1.615	>0.05
≤4.5	3 (8.6)	1 (3.6)		0 (0.0)	2 (8.0)		2 (4.2)	2 (13.3)		
>4.5	32 (91.4)	27 (96.4)		23 (100.0)	23 (92.0)		46 (95.8)	13 (86.7)		
Hydrogen peroxide (H_2_O_2_)			0.115			2.020			2.864	>0.05
Negative	4 (11.4)	4 (14.3)		2 (8.7)	6 (24.0)		8 (16.7)	0 (0.0)		
Positive	31 (88.6)	24 (85.7)		21 (91.3)	19 (76.0)		40 (83.3)	15 (100.0)		
Leukocyte esterase			0.003			0.673			0.401	>0.05
Negative	21 (60.0)	17 (60.7)		13 (56.5)	17 (68.0)		30 (62.5)	8 (53.3)		
Positive	14 (40.0)	11 (39.3)		10 (43.5)	8 (32.0)		18 (37.5)	7 (46.7)		
Sialidase			/			/			/	/
Negative	35 (100)	28 (100)		23	25		48 (100)	15 (100)		
Positive	0 (0.0)	0 (0.0)		0 (0.0)	0 (0.0)		0 (0.0)	0 (0.0)		
Lactobacillus count			0.064			0.034			0.487	>0.05
>20	9 (25.7)	8 (28.6)		7 (30.4)	7 (28.0)		14 (29.2)	3 (20.0)		
≤20	26 (74.3)	20 (71.4)		16 (69.6)	18 (72.0)		34 (70.8)	12 (80.0)		

Bold values indicate statistical significance (P < 0.05) as per standard formatting.

### 3.7 STD in HR-HPV-positive subgroups

Within the HR-HPV-positive group, no significant differences in CT, NG, or UU positivity were observed across subgroups based on viral load, genotype, or single/multiple infection status (all P > 0.05) (**Table 11**).

**Table 11:** Comparison of sexually transmitted pathogens within the HR-HPV-positive group according to viral load, genotype, and infection status [n (%)].

Pathogen	High viral load (n = 24)	Low viral load (n = 8)	χ^2^	Common genotypes (n = 11)	Other genotypes (n = 10)	χ^2^	Single infection (n = 21)	Multiple infection (n = 11)	χ^2^	P value
Chlamydia trachomatis (CT)			1.103			0.286			1.734	>0.05
Negative	21 (87.5)	8 (100.0)		9 (81.8)	9 (90.0)		18 (85.7)	11 (100.0)		
Positive	3 (12.5)	0 (0.0)		2 (18.2)	1 (10.0)		3 (14.3)	0 (0.0)		
Neisseria gonorrhoeae (NG)			/			/			/	/
Negative	21 (100.0)	8 (100.0		11 (100)	10 (100)		21 (100)	11 (100)		
Positive	0 (0.0)	0 (0.0)		0 (0.0)	0 (0.0)		0 (0.0)	0 (0.0)		
Ureaplasma urealyticum (UU)			0.549			1.014			0.286	>0.05
Negative	6 (25.0)	1 (12.5)		3 (27.3)	1 (10.0)		4 (19.0)	3 (27.3)		
Positive	18 (75.0)	7 (87.5)		8 (72.7)	9 (90.0)		17 (81.0)	8 (72.7)		

Bold values indicate statistical significance (P < 0.05) as per standard formatting.

### 3.8 Multivariable logistic regression analysis

To assess independent predictors of HR-HPV positivity, we performed multivariable logistic regression. Given the varying availability of data across parameters, two models were constructed:

- Model 1 included age (>50 vs. ≤50 years), vaginal pH (>4.5 vs. ≤4.5), and Lactobacillus count (≤20 vs. >20) as independent variables, using the 180 patients with complete data for these parameters (see **Table 8**).
- Model 2 additionally incorporated Ureaplasma urealyticum (UU) positivity (yes vs. no) and was restricted to the 76 patients with complete STD testing results plus the above variables.

The results are summarized in **Table 12**.

**Table 12:** Multivariable logistic regression analysis of factors associated with HR-HPV positivity.

Variable	Model 1 (n = 180)		Model 2 (n = 76)	
	aOR (95% CI)	P value	aOR (95% CI)	P value
Age >50 years (vs.≤50)	1.95 (1.08-3.52)	0.026	2.11 (1.02-4.36)	0.044
pH >4.5 (vs. ≤4.5)	1.63 (0.82-3.24)	0.162	1.47 (0.62-3.48)	0.384
Lactobacillus count ≤20 (vs. >20)	1.48 (0.91-2.41)	0.116	1.31 (0.66-2.60)	0.441
UU positive (vs. negative)	–	–	2.89 (1.34-6.23)	0.006

aOR, adjusted odds ratio; CI, confidence interval. Model 1 adjusted for age, pH, and Lactobacillus count. Model 2 adjusted for age, pH, Lactobacillus count, and UU status. The reduction in sample size for Model 2 reflects the availability of complete STD testing results.

In Model 1, age >50 years was significantly associated with HR-HPV positivity (adjusted OR [aOR] = 1.95, 95% CI: 1.08–3.52, P = 0.026). pH >4.5 and Lactobacillus count ≤20 showed positive associations but did not reach statistical significance after adjustment (P > 0.05).

In Model 2, after additionally adjusting for UU status, age >50 remained a significant predictor (aOR = 2.11, 95% CI: 1.02–4.36, P = 0.044). UU positivity emerged as a strong independent factor associated with HR-HPV positivity (aOR = 2.89, 95% CI: 1.34–6.23, P = 0.006). pH >4.5 and low Lactobacillus count were not significantly associated with HR-HPV in this smaller subsample.

These findings indicate that both age >50 years and UU co−infection are independently associated with HR-HPV positivity, supporting the need to account for these confounders when interpreting vaginal microecological parameters.

## 4 Discussion

This study enrolled 330 gynecological outpatients at Wusong Central Hospital, Baoshan District, Shanghai, between December 25, 2025, and February 6, 2026, to investigate associations among HR-HPV genotype, viral load, culturable bacterial richness, and STD pathogens, and to characterize changes in vaginal microecological parameters associated with HR-HPV infection. The results showed that women >50 years of age had the highest HR-HPV positivity rate, and single infections were predominantly with HPV52, HPV53, and HPV16. HR-HPV-positive patients exhibited significantly higher culturable bacterial richness indices than negative controls, and among positive patients, those with high viral load had significantly higher Ace, Sobs, and Shannon indices. HR-HPV infection was associated with marked vaginal dysbiosis and a significantly higher UU positivity rate, whereas no significant differences in microecological or STD parameters were observed within the HR-HPV-positive group across subgroups defined by viral load, genotype, or single/multiple infection status. The following sections discuss these findings in the context of recent domestic and international literature. After adjusting for age in multivariable logistic regression, UU positivity remained significantly associated with HR-HPV infection (adjusted OR = 2.89, 95% CI: 1.34–6.23, P = 0.006), whereas pH >4.5 and low Lactobacillus count were no longer independent predictors.

### 4.1 Clinical characteristics of HR-HPV infection

The overall HR-HPV positivity rate in this study was 43.94% (145/330). Single infections accounted for 73.10% (106/145), and multiple infections for 26.90% (39/145). The predominant genotypes in single infections were HPV52 (22.64%), HPV53 (11.32%), and HPV16 (9.43%). These findings align with HR-HPV genotype distribution patterns reported in East China. For instance, Cheng et al. reported that HPV52, HPV53, and HPV58 were the most common HR-HPV types among gynecological outpatients in Yangpu District, Shanghai, between 2020 and 2024, with single infections accounting for 71.3% and multiple infections for 28.7% (Cheng et al., 2025).

This distribution pattern differs from that observed in Western countries, where HPV31 and HPV51 are more common after HPV16 and HPV18 (Zhou et al., 2024). The high prevalence of HPV52, HPV53, and HPV58 in China may reflect regional differences in host genetic polymorphisms, HPV vaccination coverage, and screening strategies (Zhou et al., 2024). Currently, HPV vaccination in China primarily uses bivalent and quadrivalent vaccines, with limited coverage of the nonavalent vaccine. The bivalent and quadrivalent vaccines target HPV16 and HPV18 but do not protect against HPV52, HPV53, or other high-risk types, contributing to the persistent circulation of these genotypes (World Health Organization, 2025).

HR-HPV positivity increased with age, reaching 45.5% in women >50 years, significantly higher than in those <30 years (15.2%) or 30~50 years (39.3%). This age-related increase may be explained by postmenopausal declines in estrogen levels, leading to vaginal mucosal atrophy, reduced local immunity, decreased Lactobacillus abundance, and increased susceptibility to microecological disruption. These changes may facilitate HR-HPV acquisition and impair viral clearance, favoring persistent infection (Zeng et al., 2025). In addition, lower awareness of and participation in HPV screening among older women may contribute to the higher positivity rate in this age group (Bergengren et al., 2019).

Multiple infections accounted for 26.90% of HR-HPV-positive cases, with only 15.38% involving HPV16. This finding is consistent with a study by Spinillo et al., which suggested that HPV16 may exert negative interactions with many HR-HPV types (e.g., HPV33, HPV51, HPV52), reducing their co-infection rates. However, HPV16 exhibited a positive interaction with HPV18, increasing the risk of co-infection (Spinillo et al., 2020). The most common multiple infection combinations involved HPV52, HPV53, and HPV66. Although these combinations may have lower oncogenic potential than single HPV16 infection, they are associated with increased CIN risk compared with HPV-negative or low-risk HPV infections. Certain combinations, such as HPV52 with HPV58, may even elevate the risk of high-grade lesions (Wang et al., 2025b).

### 4.2 Association between culturable bacterial richness and HR-HPV infection

The Sobs index (species richness) was significantly higher in the HR-HPV-positive group than in the negative group (P < 0.05), indicating a greater variety of culturable microbial taxa in HR-HPV-positive patients, with lactobacilli being replaced by opportunistic pathogens such as Gardnerella, Staphylococcus, and Mycoplasma. This result is consistent with a study by Liu et al., who used 16S rRNA sequencing to demonstrate higher vaginal microbial richness in women with persistent HR-HPV infection compared to uninfected women (Liu et al., 2025). In healthy women, the vaginal microecology is characterized by low diversity and high stability, with Lactobacillus dominance. Lactobacilli maintain an acidic pH (3.8–4.5) and produce hydrogen peroxide and bacteriocins that inhibit pathogen growth, forming a crucial biological barrier. When diversity increases, lactobacilli lose dominance, the microbial community structure becomes disordered, and this state of “vaginal dysbiosis” serves as an important risk factor for HR-HPV infection (Tsementzi et al., 2025).

Patients with high viral load (logarithmic value >4) had significantly higher Sobs index than those with low viral load (P < 0.05), suggesting that increased vaginal diversity is closely linked to both HR-HPV infection and viral load. These findings are consistent with a study by Wang Pan et al (Wang et al., 2025a). The underlying mechanisms may involve pH elevation due to lactobacilli reduction, which impairs cervical epithelial barrier function and facilitates HPV adhesion and entry. In parallel, overgrowth of diverse bacteria may trigger low-grade inflammation, releasing inflammatory cytokines such as IL-6 and TNF-α, which suppress cellular immunity and reduce NK cell-mediated killing of HPV-infected cells, thereby contributing to viral persistence and high viral load (Žukienė et al., 2025).

Within the HR-HPV-positive group, no significant differences in diversity indices were observed between single and multiple infections or between common and other genotypes (P > 0.05). This suggests that increased vaginal diversity is primarily associated with HR-HPV infection per se, rather than with specific genotypes or infection multiplicity. Once infection occurs, it may be associated with microecological shifts, but the directionality remains unclear.

### 4.3 Impact of abnormal vaginal microecological parameters on HR-HPV infection

The proportions of patients with pH > 4.5, positive H_2_O_2_, positive leukocyte esterase, Lactobacillus count ≤20, and cleanliness grade III–IV were significantly higher in the HR-HPV-positive group than in the negative group (all P < 0.05), whereas sialidase positivity did not differ significantly. These findings indicate that HR-HPV infection is associated with typical features of vaginal dysbiosis, particularly reduced Lactobacillus abundance and local inflammation, consistent with multiple previous studies (Lin et al., 2022). Vaginal pH is a key indicator of microecological balance; pH > 4.5 was observed in 93.7% of HR-HPV-positive patients, significantly higher than the 80.3% in the negative group, indicating severe disruption of the acidic environment. Elevated pH can compromise cervical epithelial integrity, increase expression of HPV receptors, and reduce viral clearance, while also may promoting overgrowth of opportunistic pathogens. The higher pH and lower Lactobacillus in older women may be partly attributable to postmenopausal status, as 89% of HPV-positive women >50 were postmenopausal, a state associated with estrogen decline and vaginal atrophy.

H_2_O_2_ positivity directly reflects reduced Lactobacillus quantity and activity. In this study, 87.3% of HR-HPV-positive patients were H_2_O_2_-positive, significantly higher than the negative group. A study by Dai Youxia et al. reported that reduced Lactobacillus abundance is an independent risk factor for HR-HPV infection, with infection rates of 15.6% when Lactobacillus counts were >20 and 48.9% when counts were ≤20 (Mei et al., 2022).

It is important to note that age and menopausal status may confound the relationship between vaginal parameters and HR-HPV. In our cohort, 89.4% of HR-HPV-positive women over 50 years were postmenopausal. Estrogen decline after menopause leads to vaginal epithelial thinning, reduced glycogen availability, and decreased Lactobacillus colonization, which may independently elevate pH and lower Lactobacillus counts. Thus, the observed associations between pH, Lactobacillus, and HR-HPV should be interpreted with caution, as they may partially reflect age-related physiological changes rather than a direct causal role of microecological factors.

Leukocyte esterase positivity, indicative of local inflammation, was significantly higher in the HR-HPV-positive group (39.7%) than in the negative group (21.4%). Similarly, the proportion of patients with cleanliness grade III–IV was significantly higher in the positive group (47.6% vs. 30.8%). The lack of difference in sialidase positivity may reflect the relatively small sample size. Within the HR-HPV-positive group, no significant differences in routine vaginal parameters were observed across subgroups based on viral load, genotype, or single/multiple infection status (P > 0.05), suggesting that these parameters reflect the presence of HR-HPV infection rather than its specific virologic characteristics.

### 4.4 Association between STD pathogens and HR-HPV infection

UU positivity was significantly higher in the HR-HPV-positive group (78.1%) than in the negative group (52.3%) (P < 0.05), whereas CT and NG positivity did not differ significantly. This finding indicates a significant positive association between UU and HR-HPV, consistent with multiple domestic and international studies (Huang et al., 2024; Kristensen et al., 2025; Peng et al., 2025). UU is a commensal organism in the female urogenital tract but can overgrow and cause infection when the vaginal microecology is disrupted, further exacerbating dysbiosis and creating conditions favorable for HPV infection.

Our multivariable analysis further confirmed that this association is independent of age, with UU-positive women having nearly threefold higher odds of HR-HPV positivity (adjusted OR = 2.89, 95% CI: 1.34–6.23, P = 0.006) (**Table 12**). CT and NG positivity rates did not differ significantly between groups and were generally low (CT: 6.8%; NG: 0%), likely reflecting successful STD control measures and appropriate antibiotic use in recent years. Within the HR-HPV-positive group, no significant differences in UU, CT, or NG positivity were observed across subgroups (P > 0.05), suggesting that STD pathogen presence is primarily linked to HR-HPV infection rather than to specific viral characteristics. Clinically, routine UU testing should be considered for HR-HPV-positive patients, and those with positive UU may benefit from timely antimicrobial treatment to restore microecological balance, although prospective studies are needed to confirm whether this improves HR-HPV clearance.

The concomitant presence of UU and HR-HPV may also be associated with cervical lesion progression. Several studies have reported that co-infected women have higher CIN incidence and faster lesion progression than those with HR-HPV infection alone (Zhai et al., 2021; Zhang et al., 2025).

### 4.5 Study innovations

This study has several notable strengths. First, it provides updated data on HR-HPV genotype distribution in gynecological outpatients in the Wusong area of Shanghai, showing that HPV52, HPV53, and HPV16 are the most common types and that positivity is highest among women over 50 years. These findings may inform regional screening strategies and vaccine development efforts in China. Second, this study quantitatively evaluates the relationship between HR-HPV viral load and culturable bacterial richness, demonstrating that higher viral load is associated with greater diversity. This extends previous studies that focused primarily on the presence or absence of HPV infection. Third, the study confirms a significant association between UU and HR-HPV, supporting the inclusion of UU testing in routine management of HR-HPV infection. Fourth, the use of MALDI-TOF MS for bacterial identification provides a rapid, accurate, and high-throughput method suitable for clinical laboratories, enhancing the reliability and reproducibility of diversity analysis.

### 4.6 Limitations and future directions

Several limitations should be acknowledged. First, the study was conducted at a single center with a relatively small sample size, and the findings may not be generalizable to other populations. Second, diversity analysis was based on culturable bacteria and an equal-abundance assumption, which precludes assessment of species evenness or quantitative abundance; thus only species richness (Sobs) is reported. Third, the cross-sectional design precludes causal inference regarding the directionality of associations between vaginal microecology and HR-HPV infection. Fourth, due to incomplete data for some participants, the analyses of vaginal parameters and STD pathogens were based on a subset of the total sample, which may limit statistical power and generalizability. The retrospective nature of vaginal and STD data resulted in variable sample sizes; although missingness appeared random, the reduced sample size for STD analysis limits statistical power. Moreover, our diversity analysis was based on culturable bacteria and an equal-abundance assumption, which precludes assessment of species evenness or quantitative abundance. Consequently, we reported only species richness (Sobs index) in the main manuscript, and the Shannon/Simpson indices were relegated to **Supplementary Material** with appropriate caveats. Future studies employing metagenomic sequencing are needed to resolve true diversity dynamics.

Future studies should incorporate large, multicenter, prospective designs, using high-throughput techniques such as metagenomic sequencing and metabolomic profiling to elucidate the causal relationships and mechanistic pathways linking vaginal microecology to HR-HPV infection. Longitudinal follow-up of HR-HPV-uninfected women and those with HR-HPV infection will help clarify the temporal dynamics of microecological changes and their impact on viral clearance, persistence, and lesion progression. Ultimately, such research may support the development of targeted interventions to restore vaginal microecological balance and reduce the burden of HPV-related cervical disease.

## 5 Conclusion

This study systematically evaluated the relationships among HR-HPV genotype, viral load, culturable bacterial richness, routine vaginal parameters, and STD pathogens in 330 gynecological outpatients. The results suggest that HR-HPV infection is associated with significant vaginal microecological dysbiosis, characterized by increased bacterial richness (Sobs index), elevated pH, reduced Lactobacillus abundance, and local inflammation. The degree of dysbiosis is correlated with viral load, with higher viral load associated with more pronounced alterations. UU co-infection is positively associated with HR-HPV infection, whereas no significant associations were observed for CT or NG. HR-HPV infection was more common in women over 50 years of age, and single infections were predominantly with HPV52, HPV53, and HPV16.

These findings support the integration of vaginal microecological assessment and UU screening into adjunctive strategies for HR-HPV infection management. However, causality remains to be established in prospective studies. For women with evidence of microecological dysbiosis, intensified HR-HPV screening is warranted. For HR-HPV-positive patients, routine evaluation of vaginal microecology and UU status may facilitate timely interventions to restore microecological balance, improve HR-HPV clearance, and reduce the risk of persistent infection and cervical lesion progression. Future large-scale, multicenter, prospective studies are needed to clarify the causal relationship and underlying mechanisms linking vaginal microecology and HR-HPV infection, thereby providing a stronger evidence base for comprehensive prevention strategies.

## Data Availability

The original contributions presented in the study are included in the article/**Supplementary Material**. Further inquiries can be directed to the corresponding author.

## Supplementary material

Supplementary Table 1 Shannon and Simpson indices (raw data) in different groups [M (Q1, Q3)].

Supplementary Table 2 Comparison of key characteristics between included and excluded patients.
