# Supplementary material for: Vaginal microecological diversity and Ureaplasma urealyticum co-infection in high-risk HPV infection: a cross-sectional study
Source: Front Cell Infect Microbiol. 2026 Jun 29;16:1851005. doi: 10.3389/fcimb.2026.1851005 (PMC13357797; doi:10.3389/fcimb.2026.1851005)
Supplement: Supplementary Table 1 — Shannon and Simpson indices (raw data) in different groups [M (Q1, Q3)]. [file Table1.docx]

**Supplementary Material**

**Supplementary Table S1. Shannon and Simpson indices (raw data) in different groups [M (Q1, Q3)]**

Due to the equal‑abundance assumption (each detected species counted as 1), Shannon and Simpson indices mathematically reflect presence/absence rather than true diversity; these results should be interpreted with caution.

| **Comparison** | **Group** | **n** | **Shannon index** | **Simpson index** |
| --- | --- | --- | --- | --- |
| HR‑HPV negative vs. positive | Negative | 185 | 1(0, 1.6) | 0.7(0.5, 1.0) |
|  | Positive | 145 | 1(1.0, 1.6) | 0.7(0.5, 0.8) |
| P value |  |  | < 0.001 | 0.010 |
| Viral load (positive group) | Low | 55 | 1(1.0,1.6) | 0.7 (0.5, 0.8) |
|  | High |  | 1(1.0,2.0) | 0.7 (0.5, 0.8) |
| P value |  |  | 0.027 | 0.568 |
| Genotype (single infection) | Common | 58 | 1(1.0,1.6) | 0.7 (0.5, 0.8) |
|  | Other | 48 | 1.6(1.0,1.6) | 0.7 (0.5, 0.8) |
| P value |  |  | 0.432 | 0.101 |
| Infection multiplicity | Single | 106 | 1(1.0,1.6) | 0.7 (0.5, 0.8) |
|  | Multiple | 39 | 1(1.0,2.0) | 0.7 (0.5, 0.8) |
| P value |  |  | 0.968 | 0.489 |

**Note:** All comparisons used Mann–Whitney U test. Shannon and Simpson indices are presented here for completeness but were not used for statistical inference in the main manuscript due to the equal‑abundance limitation.

**Supplementary Table S2. Comparison of key characteristics between included and excluded patients**

| **Analysis** | **Characteristic** | **Included** | **Excluded** | **P value** |
| --- | --- | --- | --- | --- |
| A. Vaginal discharge analysis | n | 180 | 150 | - |
|  | Age, median (IQR), years | 46 (34-57) | 45 (33-56) | 0.342 |
|  | HR-HPV positive, n (%) | 63 (35.0%) | 82 (54.7%) | < 0.001 |
|  | HPV16/18 positive (among HR-HPV+), n (%) | 13 (20.6%) | 15 (18.3%) | 0.713 |
| B. STD pathogen analysis | n | 76 | 254 | - |
|  | Age, median (IQR), years | 44 (33-55) | 46 (34-58) | 0.189 |
|  | HR-HPV positive, n (%) | 32 (42.1%) | 113 (44.5%) | 0.706 |
|  | HPV16/18 positive (among HR-HPV+), n (%) | 5 (15.6%) | 23 (20.4%) | 0.543 |

**Notes:**

- For vaginal discharge analysis, included patients were those with complete data on pH, H₂O₂, leukocyte esterase, sialidase, Lactobacillus count, and cleanliness grade; excluded patients lacked one or more of these parameters.
- For STD analysis, included patients had complete CT, NG, and UU test results; excluded patients lacked STD testing.
- P values: Age compared by Mann–Whitney U test; proportions compared by χ² test.
- The higher proportion of HR-HPV positivity in excluded patients for vaginal discharge analysis likely reflects that clinicians were more likely to order discharge tests when infection was suspected; however, among HR-HPV-positive patients, HPV16/18 distribution did not differ, suggesting no severe selection bias for genotype-specific analyses.
